# National implementation of HPV vaccination programs in low-resource countries: Lessons, challenges, and future prospects

**DOI:** 10.1016/j.ypmed.2020.106335

**Published:** 2021-03

**Authors:** Vivien D. Tsu, D. Scott LaMontagne, Phionah Atuhebwe, Paul N. Bloem, Cathy Ndiaye

**Affiliations:** a University of Washington, Seattle, USA; b Center for Vaccine Innovation and Access, PATH, Seattle, USA; c Africa Regional Office, World Health Organization, Brazzaville, Congo; d Life Course and Integration/EPI, Department of Immunization, Vaccines and Biologicals, World Health Organization, Geneva, Switzerland; e PATH, Dakar, Senegal

**Keywords:** HPV vaccination, Implementation, Low and middle-income countries, National introduction

## Abstract

More than 90% of cervical cancer deaths occur in low- and middle-income countries (LMICs), which have limited capacity to mount the comprehensive national screening and precancer treatment programs that could prevent most of these deaths. The development of vaccines against the human papillomavirus (HPV) has dramatically altered the landscape of cervical cancer prevention. As of mid-2020, 56 LMICs (41% of all LMICs) have initiated national HPV vaccination programs. This paper reviews the experience of LMICs that have introduced HPV vaccine into their national programs, key lessons learned, HPV vaccination sustainability and scale-up challenges, and future mitigation measures.

As international guidance evolved and countries accumulated experience, strategies for national introduction shifted with regard to target groups, delivery site and timing, preparation and planning, communications and social mobilization, and ultimately monitoring, supervision and evaluation. Despite the successes that LMICs have been able to achieve in reaching large proportions of eligible girls, there are still considerable challenges countries encounter in overcoming rumors, reaching out-of-school girls, completing the vaccine series, estimating target populations, monitoring program performance, and assuring vaccination sustainability. New opportunities, such as the entry of additional vaccine manufacturers and ongoing studies to evaluate one-dose delivery, could help overcome the outstanding barriers to higher coverage and financial sustainability. Effective use of the experience to date and advances on the horizon could enable all LMICs to move towards the coverage levels that are needed to achieve eventual elimination.

## Highlights

• School-based delivery achieves higher coverage but countries concerned about cost.
• Trade-offs between age- and grade-based strategy remains complex, country-specific.
• Managing rumors and misinformation is critical to success; preparedness is key.
• Good coordination between health and education sectors essential for HPV vaccine.

## 1 Background

Cervical cancer causes more than 300,000 deaths each year and is the leading cause of cancer death among women in Sub-Saharan Africa (Arbyn et al., 2020), even though it is almost completely preventable. More than 90% of the deaths occur in low- and middle-income countries (LMICs) (International Agency for Research on Cancer, 2019), which have limited capacity to mount comprehensive national screening and precancer treatment programs that have effectively reduced cervical cancer incidence and mortality rates in wealthier countries (Ogilvie et al., 2017). The development of vaccines against the human papillomavirus (HPV), which is the primary cause of cervical cancer, has dramatically altered the landscape of cervical cancer prevention and led in 2018 to the call by the World Health Organization (WHO) to eliminate the disease globally (WHO, 2018). Since licensure of the first HPV vaccine in 2006, its effectiveness in preventing the precursors to cervical cancer (infection and lesions) has surpassed expectations (de Sanjose et al., 2019), and updated simulation models suggest that the greatest benefit will be seen in Africa, given its heavy disease burden (exacerbated by HIV), population growth, and increase in life expectancy (Abbas et al., 2020).

HPV vaccination using a 3-dose schedule was recommended to countries by WHO in 2009 (WHO, 2009) and was approved for support to eligible LMICs by Gavi, the Vaccine Alliance in 2011 (Gavi, the Vaccine Alliance (Gavi), 2011). Based on new evidence, WHO updated its guidance on the schedule in 2014 to recommend a 2-dose regimen for girls aged 9–14 years (WHO, 2014a) and updated it again in 2017 to recommend that countries consider vaccinating a multi-age cohort (MAC), instead of a single cohort, at first introduction of the vaccine to accelerate the impact and improve program efficiency (WHO, 2017). With constrained HPV vaccine supply becoming evident in 2018, SAGE recommended in 2019 a temporary pause in MAC vaccinations and even considered the possibility of an extended interval of 2–3 years between the first and second doses for countries currently vaccinating and experiencing supply constraints (WHO, 2019).

**Fig. 1:**
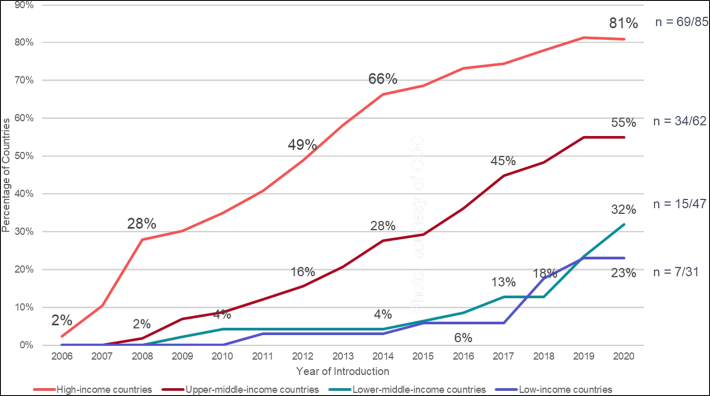
HPV vaccine introductions by World Bank classifications, 2006 - June 2020.

Over the course of this evolving policy landscape, LMICs began introducing the vaccine into their national immunization programs, with varying experiences–some after donation-based pilot programs or Gavi-funded demonstration projects and some without prior experience (Gallagher et al., 2018). As of mid-2020, 56 LMICs (41% of all LMICs) had initiated national HPV vaccine programs (see Table 1) (PATH, 2020), and 9 more have been approved by Gavi (as of May 2020) for vaccine support as supplies become available (Gavi, 2020a). The situation is fluid, with additional countries introducing the vaccine each month (Cameroon, El Salvador, and Myanmar, which introduced in the second half of 2020, are not included in the rest of the paper). While the pace of introductions in high and upper-middle income countries rose steadily from 2006, adoption in low and lower-middle income countries did not take off until 2017 (LaMontagne et al., 2017), and 11 of these programs were initiated in 2019 or 2020 (Fig. 1).

**Table 1:** Summary characteristics of national HPV vaccination programs in LMICs (as of June 2020).

Country	WHO region	World Bank category (2019)	Year of introduction	Target group for vaccination	Primary delivery strategy	Dosing schedule	Gavi-supported	HPV1% (2019)	HPVc % (2019)
Ethiopia	AFR	LIC	2018	14-year-old girls	Schools	0,6 months	Yes	94	84
Gambia	AFR	LIC	2019	9- to 14-year-old girls (1st year); 9-year-old girls (subsequent years)	Schools	0,12 months	Yes	68	n/a
Liberia	AFR	LIC	2019	9-year-old girls	Mixed (schools + outreach)	0,6 months	Yes	14	n/a
Malawi	AFR	LIC	2019	9-year-old girls	Schools	0,6 months	Yes	88	n/a
Rwanda	AFR	LIC	2011	12-year-old girls	Schools	0,6 months	Yes	97	94
Tanzania	AFR	LIC	2018	14-year-old girls	Health facilities	0,6 months	Yes	78	49
Uganda	AFR	LIC	2015	10-year-old girls	Mixed (schools + child health days)	0,6 months	Yes	99	64
Bhutan	SEAR	LMIC	2010	12-year-old girls	Schools	0,6 months	No	89	73
Bolivia	AMR	LMIC	2017	10-year-old girls	Schools	0,6 months	Yes	88	80
Côte d'Ivoire	AFR	LMIC	2019	9-year-old girls	Mixed (schools + outreach)	0,6 months	Yes	6	n/a
Honduras	AMR	LMIC	2016	11-year-old girls	Schools	0,6 months	Yes	78	59
Indonesia^b^	SEAR	LMIC	2019	11- to 12-year-old girls	Schools	0,6 months	No	n/a	0.5
Kenya	AFR	LMIC	2019	10-year-old girls	Mixed (schools + outreach)	0,6 months	Yes	25	n/a
Lao, People's Democratic Republic^a^	WPR	LMIC	2020	10- to 14-year-old girls (1st year); 10-year-old girls (subsequent years)	Schools^c^	0,12 months	Yes	n/a	n/a
Federated States of Micronesia^d^	WPR	LMIC	2009	10- to 11-year-old girls in grade 5	Schools	0,6 months	No	70	57
Moldova	EUR	LMIC	2020	10-year-old girls	Health facilities	0,6 months	No	n/a	31
Philippines^b^	WPR	LMIC	2015	Girls in grade 4 (~9 years old)	Schools	0,6 months	No	7	0.2
Senegal	AFR	LMIC	2018	9-year-old girls	Health facilities	0,6 months	Yes	86	25
Solomon Islands	WPR	LMIC	2019	9- to 14-year-old girls (1st year); 9-year-old girls (subsequent years)	Schools	0,12 months	Yes	67	n/a
Uzbekistan	EUR	LMIC	2019	9-year-old girls	Schools	0,6 months	Yes	97	n/a
Zambia	AFR	LMIC	2019	14-year-old girls	Schools	0,12 months	Yes	99	n/a
Zimbabwe	AFR	LMIC	2018	10 to 14-year-old girls (first year); grade 5 (subsequent years)	Schools	0,12 months	Yes	91	67
Argentina	AMR	UMIC	2011	11-year-old girls and boys	Health facilities	0,6 months	No	84	57
Armenia	EUR	UMIC	2018	13-year-old girls	Health facilities	0,6 months	No	17	7
Belize	AMR	UMIC	2016	9-year-old girls	Schools	0,6 months	No	99	89
Botswana	AFR	UMIC	2015	Girls in grade 5	Schools	0,6 months	No	87	47
Brazil	AMR	UMIC	2014	9- to 14-year-old girls and 11- to 14-year old boys	Health facilities	0,6 months	No	83	69
Bulgaria	EUR	UMIC	2012	12- to 13-year-old girls	Health facilities	0,6 months	No	7	5
Colombia	AMR	UMIC	2012	9- to 17-year-old girls	Schools	0,6 months	No	71	39
Cook Islands^e^	WPR	UMIC	2011	9-year-old girls	Schools		No	n/a	n/a
Costa Rica	AMR	UMIC	2019	10-year-old girls	Schools	0,6 months	No	98	39
Dominica	AMR	UMIC	2019	11- to 12-year-old girls and boys	Schools	0,6 months	No	100	n/a
Dominican Republic	AMR	UMIC	2017	9-year-old girls	Schools	0,6 months	No	12	7
Ecuador	AMR	UMIC	2014	9-year-old girls	Mixed (schools + health facilities)	0,6 months	No	82	54
Fiji	WPR	UMIC	2013	9- to 13-year-old girls	Schools	0,6 months	No	93	56
North Macedonia	EUR	UMIC	2009	12-year-old girls	Schools	0,6 months	No	45	40
Georgia^b^	EUR	UMIC	2019	10- to 12-year-old girls	Health facilities	0,6 months	No	38	11
Grenada	AMR	UMIC	2019	9- to 10-year-old girls	Schools	0,6 months	No	73	41
Guatemala	AMR	UMIC	2018	10-year-old girls	Schools	0,6 months	No	42	24
Guyana	AMR	UMIC	2017	9- to 14-year-old girls and boys	Schools	0,6 months	Yes	31	13
Jamaica	AMR	UMIC	2017	Girls in grade 7 (11- to 12-year-old girls)	Schools	0,6 months	No	23	9
Libya^e^	EMR	UMIC	2014	12-year-old girls	Schools	0,6 months	No	n/a	n/a
Malaysia	WPR	UMIC	2010	13-year-old girls	Schools	0,6 months	No	86	85
Maldives	SEAR	UMIC	2019	10-year-old girls	Schools	0,6 months	No	88	n/a
Marshall Islands	WPR	UMIC	2009	Girls in grade 6 (11- to 12-year-old girls)	Schools	0,6 months	No	67	24
Mauritius	AFR	UMIC	2016	9-year-old girls	Schools	0,6 months	No	84	80
Mexico	AMR	UMIC	2012	9- to 11-year-old girls	Mixed (schools + outreach)	0,6 months	No	94	95
Paraguay	AMR	UMIC	2013	10-year-old girls	Schools	0,6 months	No	70	61
Peru	AMR	UMIC	2011	9- to 13-year-old girls (grade 5 if in school)	Schools	0,6 months	No	82	76
Saint Lucia	AMR	UMIC	2019	11- to 12-year-old girls and boys (grade 6)	Schools	0,6 months	No	86	46
Saint Vincent and the Grenadines	AMR	UMIC	2017	Girls in grade 6 (11- to 12-year-old girls)	Schools	0,6 months	No	n/a	10
South Africa	AFR	UMIC	2014	9-year-old girls (grade 4)	Schools	0,6 months	No	69	56
Sri Lanka	SEAR	UMIC	2017	Grade 6 (~10-year-old girls)	Schools	0,6 months	Yes	99	82
Suriname	AMR	UMIC	2013	9- to 13-year-old girls	Schools	0,6 months	No	51	38
Thailand	SEAR	UMIC	2017	11-year-old girls	Schools	0,6 months	No	76	66
Turkmenistan	EUR	UMIC	2016	9-year-old girls and boys	Health facilities	0,6 months	No	n/a	99

Program data: compiled from multiple sources, including peer-reviewed publications, national government websites and reports, global databases, and international organizations.

Coverage data: extracted from Bruni et al., 2021 (https://doi.org/10.1016/j.ypmed.2020.106399).

a Recent introduction in 2020.

b Sub-national introduction.

c Due to COVID and school closures, extra focus on facility based and community outreach at introduction.

d Follows vaccine policy of the United States of America.

e Coverage data not available in Bruni et al., 2021.

Drawing on the published literature, program reports, and the observations of the authors relevant to HPV immunization programs, this paper reviews the experience of LMICs that have introduced HPV vaccination into their national programs: key lessons learned, HPV vaccination sustainability and scale-up challenges, and future mitigation measures. Since the majority of the introductions have been relatively recent, the few publications available mostly reflect only the first year of introduction. Post introduction evaluations (PIEs) have been carried out in at least 15 countries, and many countries have carried out national meetings after the first or second dose to assess successes and challenges, but the findings from these are not always published or publicly available.

## 2 Experience to date and main lessons learned

Although data are not available from all 56 LMICs that have introduced HPV vaccine, past reviews have shown relative consistency across countries in the experiences and best practices that have emerged in some program elements, while unanswered questions remain in other areas (Gallagher et al., 2017). As international guidance evolved and as countries tried various approaches in pilot and demonstration projects, strategies for national introduction shifted with regard to target groups, delivery strategy, preparation and planning, communications and social mobilization, and ultimately monitoring, supervision, and evaluation.

### 2.1 Target population

Most countries began introduction with a single age or school-grade cohort of girls aged between 9 and 11 years, based primarily on the convenience of finding high rates of school enrollment at that age and on the concern that sexual debut might occur soon after this age for some girls. After the temporary vaccine supply constraint (that particularly affected Gavi-eligible countries) was identified in 2018, SAGE recommended all new introductions begin with a single cohort of the oldest eligible girls who would “age out” of eligibility before more vaccine became available. At least three countries (Ethiopia, Tanzania, and Zambia) took this approach and most achieved good coverage (Bruni et al., 2021). Other countries (such as Côte d'Ivoire, Liberia, Malawi, and Senegal) decided not to start with older girls because they were not certain they could achieve good coverage with the older age group, especially where dropout between primary and secondary school is high. While identification of eligible girls by grade proved easier for school-going girls, it created problems for determining a comparable eligibility criterion for out-of-school (OOS) girls. It may be easier for the Ministry of Education to estimate the size of the target population by grade, but it is often difficult to have reliable estimates of OOS girls or to distinguish between girls enrolled and those who actually attend regularly. Determination of individual eligibility by age has its own challenges, depending on how well girls (or their families) know their ages and the level of documentation school programs and health centers require as proof of age. When school attendance is high and the age spectrum of girls in each class is relatively narrow, defining eligibility by grade can work very well when school-based strategies are used. For community or facility-based strategies or where OOS girls are a significant part of the population, age-based eligibility is usually preferable.

Gavi-eligible countries were limited in their support to a single cohort until 2016. Nine LMICs not dependent on Gavi support (Belize, Botswana, Brazil, Colombia, Fiji, Peru, Philippines, Rwanda, and Suriname) provided vaccine to a catch-up group of older girls up to age 13, 15, or 18 (Gallagher et al., 2017), and six countries (Bolivia, The Gambia, Guyana, Lao PDR, Solomon Islands, and Zimbabwe) conducted MAC vaccinations in 2017–20 with Gavi support. In addition to accelerating the impact by vaccinating girls closer to the time when infection might occur (Jit and Brisson, 2018), multi-age cohorts can generate program efficiencies by spreading program costs for service delivery and social mobilization over a larger group of beneficiaries (Gallagher et al., 2017). MACs can, however, also increase costs (and lower coverage) if older girls require outreach to secondary schools, have lower school attendance rates, or are less likely than younger girls to come to facilities or community outreach services. Some countries (Bolivia, Zimbabwe) did not report lower coverage for older ages, but others like Rwanda did report difficulties reaching older cohorts (Sayinzoga et al., 2020).

### 2.2 Delivery strategy

Countries must make several critical decisions about vaccine delivery, including the primary and any secondary vaccination sites, the timing and duration of delivery, and whether delivery will be integrated with other health or community services. Most countries have selected schools as the primary vaccination site at least initially, either with special outreach campaigns or as part of existing school health programs [17; 20], but strategies have also shifted over time and have varied within countries. These have usually been complemented with facility and routine community outreach services as secondary sites for OOS girls or school girls who missed a dose at school, either on the original visit or subsequent mop-up visits. However, where school visits are not already part of the immunization program budget, some countries have decided the costs of school-based delivery of HPV vaccine are too high and they instead offer the vaccine at health facilities along with other routine immunization services and rely on social mobilization campaigns to inform and motivate girls and their families to attend. Initial coverage is generally much lower than school-based programs (Bruni et al., 2021; Brotherton and Bloem, 2018). While other new vaccines have experienced increasing coverage over time after introduction, early analyses have suggested that the initial level of coverage for HPV vaccine achieved in the early years of the program tends to be a strong predicter of subsequent levels of coverage over time (Bruni et al., 2021).

Unlike infant vaccines, there is more flexibility about the timing during the calendar year and duration of provision of the required two-dose schedule of HPV vaccines. The vaccine can be administered on a semi-annual (0, 6 months) or annual schedule (0, 12 months) and can be done as part of a concentrated campaign or on a continuous basis, available throughout the year. A concentrated campaign approach allows for a focused communication strategy with teacher education, school messages, and mass media, while a continuous schedule approach (used more often with facility or community-based strategies) may require more ongoing messaging or activity by health workers or community agents. Senegal, for example, chose a continuous schedule with monthly visits to schools and girls vaccinated as they reached the eligible age; they considered this more feasible and sustainable. Campaigns can be compressed into a few days or spread over a month to allow for regional adaptation, depending on coordination with the schedules of other activities and the degree of work disruption that vaccination teams can tolerate. Careful consideration of the timing of school-based delivery is essential to ensure that it does not conflict with school exams or holidays and is not hampered by rainy seasons or other barriers.

Another consideration in designing a delivery strategy for HPV vaccination is whether to integrate the activity with other health or education services. At least three LMICs (Malaysia, South Africa, and Sri Lanka) have existing school health programs into which HPV vaccine has been integrated (Mahumud et al., 2020; Delany-Moretlwe et al., 2018), and at least two countries (Uganda and Zambia) have used semi-annual or annual Child Health Days as a platform for delivering HPV vaccine. Several Latin American countries integrate HPV vaccine delivery into national immunization weeks. Adolescent health interventions for integration that might enhance synergies and program efficiency were identified by WHO (WHO, 2014b). In Tanzania, the most feasible interventions were found to be deworming, HIV, and menstrual or adolescent reproductive health education. However, stakeholders have raised concerns as to whether health workers would have time in the midst of vaccination visits to carry out these extra activities (Watson-Jones et al., 2016). Although outcomes are not yet known, countries have reported integration with Vitamin A administration (Uganda), deworming (Belize, Ecuador, Rwanda, South Africa, Uganda), iron and folic acid supplementation (Belize), health check-ups (Fiji, Malaysia, Suriname), and growth/visual/dental or oral checks (Dominica, Fiji, Guatemala, Thailand) (WHO, 2020a).

### 2.3 Preparation and planning

Countries have had considerable experience introducing new vaccines over the past decade and many of the proven best practices apply to HPV vaccine as well, but there are some differences and specific tools that have been developed for HPV vaccine. Because HPV vaccine affects an age group not previously served by immunization programs and engages a wider group of actors and sectors (such as cancer, reproductive health, education, and women's affairs), planning at national and local levels requires more time and coordination; there are also budget implications for including non-health personnel who may require training or incentives for taking on vaccination-related tasks. The national statistics unit plays an important role in estimating the size of the target population, which differs from the typical birth cohort target used for most vaccines. Countries have found it helpful to form working groups at least 6 months in advance of the planned launch, and micro-planning at the district level should include at least health and education sector representatives. WHO has prepared several HPV-specific tools for planning, including a guide to introducing the vaccine, a vaccine introduction readiness tool, a guide for planning communication, a school vaccination readiness assessment tool, and a brief on issues around consent when vaccinating children and adolescents (WHO, 2020b).

Training is a resource-intensive but essential part of the preparation for introducing a new vaccine, and this is especially true for HPV vaccine given the age group and the need to involve teachers and school leaders when school-based strategies are undertaken. Most countries have used a traditional cascade approach, training regional trainers who then go out to train at lower levels. At least one country, however, has used some innovative techniques; Lao PDR, which was the first to introduce the HPV vaccine during the COVID-19 pandemic, used recorded videos and interactive PowerPoint slides to ensure that messages were conveyed consistently at all levels (Franzel and Yang, 2020). Them has been important to tailor training materials to the various audiences and the roles that they will play; for example, teachers need to be able to identify eligible girls and to explain to parents the purpose and process of vaccination, including the need for two doses and the planned schedule. When teachers are not directly involved in vaccination programs, simple sensitization may be sufficient. Health workers need more detailed information, including about managing and reporting adverse events. Everyone working in contact with the community needs to know how to recognize rumors and misinformation and the designated channels for managing them. Many countries (such as Armenia, Georgia, Senegal, and Zimbabwe) have found it useful to provide orientation sessions and resource materials to journalists. A few countries have started using mobile phone-based payment systems to pay transport and other allowances to reduce the delays and bookkeeping burdens associated with traditional disbursement systems.

### 2.4 Communications and social mobilization

Countries have used a wide array of communications channels to inform and motivate girls, their families, and influential community gatekeepers. Key messages have been identified based on earlier experience with demonstration projects, with a focus on cancer prevention, safety of the vaccine, government endorsement, clear explanations of eligibility for vaccination and how many doses are needed, and practical information such as where it will be administered and when (Gallagher et al., 2017). Most countries have used a combination of interpersonal methods (like health talks by health workers and teacher-parent meetings), print materials (such as posters, banners, and brochures), mass media (especially radio), and social media (such as WhatsApp, Facebook, and mass text messages). Several countries have reported during PIEs that print materials were often not received in time or in sufficient quantities, but there is little evidence that this seriously hampered vaccine acceptance. Coverage surveys have provided useful feedback on which communication channels were most effective (LaMontagne et al., 2011); such guidance could be used in future to design more cost-effective social mobilization strategies, since this component is one of the most expensive elements in introduction programs (Botwright et al., 2017).

Given the controversy that often surrounds HPV vaccine, successful programs have prepared crisis response plans and identified designated spokespeople. Programs that monitored the media–and especially social media–for misinformation and mounted prompt responses were better able to prevent rumors from derailing vaccine delivery (Pan American Health Organization, 2018). For example, in Senegal all rumors and their sources were identified and listed in a table (Facebook, WhatsApp, newspapers) and were reviewed by a technical working group, which then designed a plan to respond to the most frequent issues by participating in TV and radio shows and diffusing continuous messages. In several countries (such as Bolivia and Zambia), the use of WhatsApp groups for health workers across the country has facilitated the rapid identification and addressing of rumors. Community leaders also play an important role in identifying circulating misconceptions and responding to them with correct messages. In the case of adverse events after immunization, prompt and thorough investigation as happened in Brazil is crucial for maintaining confidence and avoiding vaccine hesitance (Marchetti et al., 2020).

## 3 Ongoing challenges

Despite the successes that LMICs have been able to achieve in reaching large proportions of eligible girls—in many cases with higher coverage than in wealthier countries (particularly with first dose coverage) (Bruni et al., 2021)—there are still considerable challenges countries encounter in overcoming rumors, reaching OOS girls, completing the vaccine series, estimating target populations, monitoring program performance, and assuring sustainability. In some cases (such as how to reach HIV-positive girls with a third dose or how to reach and monitor OOS girls), solutions to these problems are not yet apparent and need further investigation, and perhaps even experimentation. However, better documentation of positive national experiences and sharing of lessons learned, especially regionally, could help other countries that are earlier in the process of introduction avoid the pitfalls and mitigate the inherent difficulties.

### 3.1 Getting the message out

A variety of factors influence both the ability of governments to inform the population and the social environment in which vaccine delivery occurs. Most countries now have a good sense of the messages that are needed, but logistical and bureaucratic obstacles often prevent materials or payments (e.g., for transport or public meetings) from being distributed in a timely way. Among ethnically diverse groups or other marginalized populations or where historical or political reasons contribute to distrust of government, there may be heightened skepticism of government messaging and greater susceptibility to rumors and misinformation. Many countries have been careful to involve faith leaders in their planning, but opposition on religious grounds has occurred on several occasions and is difficult to counter once it is established. Journalists are sometime poorly informed or may have incentives to create or amplify sensational stories about alleged vaccine side effects. The rapid spread of stories on social media (often promoted by international anti-vaccine groups) is particularly difficult to manage once it starts. Particular HPV vaccine events in specific countries can have international influence; for example, the HPV crisis in Japan influenced the African countries starting their demonstration projects in 2013–2014, and the hesitancy in France (Lahouati et al., 2020), that is particularly strong in the case of HPV vaccine, may have influenced both the Francophone African countries as well as the introduction in Armenia through the sizeable Armenian emigrant population in France.

### 3.2 Achieving high and equitable coverage and minimizing dropouts

Median estimated program coverage for the first dose (HPV1) among girls in LMICs in 2019 was 80%, but there was a nearly 20% drop in those receiving the second dose (Bruni et al., 2021). Constraints on delivery of the first dose usually reflect system barriers like confusion about eligibility, burdensome consent requirements, and insufficient outreach visits. Facility-based programs that rely on families to bring girls to clinics for immunization is estimated to be cheaper by avoiding sending teams to schools but often costs more to inform and motivate girls to attend (Gallagher et al., 2017). Several factors have been suggested as causes for the failure of girls to complete the series, including poor tracking systems, failure to reinstate social mobilization efforts or provide adequate refresher training for the second round when using campaign approaches, girls' switch to secondary school (for annual schedules), and continuous schedules where there is no particular reminder to get the second dose. Events like health worker strikes (Senegal) and the COVID-19 pandemic can also cause temporary interruptions to introductions (Cabo Verde, Cameroon, El Salvador, Lao PDR, Sao Tome), as well as ongoing programs.

### 3.3 Multisector coordination

For the majority of programs that rely on school-based delivery, coordination between health and education sectors is essential, but even within the health sector several different units need to work together to coordinate schedules and messaging. While training of teachers, informing parents, and scheduling sessions to avoid school exams and holidays (both national and religious) seem to be managed well in most countries, enumeration of eligible girls, obtaining consent where required, identifying eligible girls if age is the criterion, and recordkeeping still present challenges for many countries. Within the education sector, private schools are sometimes more difficult to coordinate with and may be less willing to cooperate; in particular, they may have more stringent consent requirements. More data and case studies are needed to determine effective solutions.

### 3.4 Determining numerators and denominators

Both numerators (the number of girls vaccinated) and denominators (the target population) are essential for planning and monitoring vaccination programs. Countries using census data to estimate the target population are plagued by out-of-date census numbers often complicated by internal migration and/or cross-border flow of migrants or refugees or by girls shifting residence for the school year; this dilemma is true for other vaccines as well and can lead to overestimates of coverage (by underestimating the denominator). Less commonly, overestimation of numerators (for example, due to age misclassification) can also lead to overestimation of coverage. When countries consult multiple sources for population data, there can be confusion about how to reconcile discrepancies. Determining denominators (targets for estimating supplies and coverage) was further complicated for countries that decided to vaccinate girls missed in earlier rounds (such as Bolivia, which increased its coverage by more than 5% through this practice). Reliance on school enrollment figures can work well for grade-based approaches but works poorly for age-based programs and also raises the risk of missing substantial numbers of OOS girls, if primary school attendance is low. Some countries choose instead to enumerate the eligible girls prior to starting vaccinations, a resource-intensive approach with its own risks of missing girls from marginalized populations. Because the WHO indicators of HPV vaccine coverage call for reporting of doses delivered by age 15, there is pressure either to adopt an age-based approach, to record age at the time of vaccination, or to “attribute” doses delivered in a grade to a specific age (Bruni et al., 2021).

### 3.5 Sustainability

The cost of the vaccine itself is the primary barrier to ultimate sustainability, whether a country pays just a portion for Gavi-subsidized vaccine, a Gavi or PAHO-guaranteed price, or full market price. Middle-income countries without access to Gavi or PAHO prices (referred to as “the missing middle”) are particularly challenged to make fiscal space in their budgets for HPV vaccine at current prices, as are countries with especially large populations (Gallagher et al., 2018). On the other hand, about half of upper-middle income countries have managed to introduce the vaccine even without Gavi support (PATH, 2020), often countries with existing school health programs or school-based vaccination. While Gavi-eligible countries have the opportunity to do one-time MAC vaccinations to catch up the vaccination of girls who did not have the opportunity to be vaccinated due to the relatively recent introduction of most HPV vaccine programs, non-Gavi countries often have to forego any catch-up campaigns. The cost of delivery is the other challenge if there is no existing school health program into which HPV vaccine can be easily integrated. Conducting outreach visits to schools once or twice a year, training teachers, informing parents, and obtaining consent where required are extra costs for school-based programs, and all programs require age-appropriate materials for young adolescents and comprehensive education and social mobilization in communities. There is an important role for advocates, both national and international, to champion the allocation of needed resources while countries continue to search for the most cost-effective approaches.

### 3.6 Vaccine supply constraints

In the next several years, LMICs can still expect supply constraints to limit their ability either to introduce the vaccine or to carry out MACs. This has affected planning for Gavi-eligible countries, many of which had to delay their MACs and shift their single-age cohorts to older girls before they age out of eligibility. This also affects non-Gavi countries as it hampers their ability to negotiate favorable prices, when they must compete against wealthier countries that are doing gender-neutral programs and serving broader age ranges.

## 4 Opportunities and future prospects

One of the most exciting recent developments came in June 2020 when Gavi announced that three new manufacturers, along with the original two, had committed to prioritizing HPV vaccine supply to Gavi that could enable up to 84 million girls in Gavi-eligible countries to receive vaccine in the next 5-year period starting 2021 (Gavi, 2020b). The new manufacturers include Innovax and Walvax of China and Serum Institute of India. Innovax has received regulatory approval in China from the National Medical Products Administration (NMPA) and its documentation for prequalification by WHO is under review – a process that is required for any vaccine to be purchased by UNICEF. Walvax has filed with the China NMPA for regulatory approval, with WHO prequalification submission expected in second quarter 2021. Serum Institute of India has not yet applied for national regulatory approval but will probably do so in 2021 and expects to apply for WHO prequalification by 2022. These vaccines should ensure both greater supply capacity and lower prices and should enable the two largest LMICs, China and India, to join the ranks of countries that are offering vaccine protection to their girls.

As more countries introduce the vaccine into their national programs using a variety of approaches to planning, delivery, communication, and monitoring, there will be more opportunities to share the lessons learned and best practices for achieving greater efficiency and coverage. Regional meetings like the ones organized in recent years by PAHO and AFRO enable immunization staff to talk with their counterparts in neighboring countries and seek practical advice on what works best (Pan American Health Organization, 2018). This growing body of knowledge should accelerate the process of program refinement, especially for those countries just starting introduction and national implementation.

There are potential scientific and manufacturing advances on the horizon that could have a significant impact on future HPV immunization programs. One of the most important is the accumulation of evidence as to whether one dose of HPV vaccine is sufficient to ensure long-term protection. Reviews of available opportunistic data have suggested the promise of this regimen and inspired the initiation of clinical trials to evaluate it more rigorously (Bergman et al., 2019; Whitworth et al., 2020). If the results confirm the efficacy of a single-dose schedule, the impact could be enormous—reducing the cost of vaccine and delivery, simplifying the logistics, and opening the door to gender-neutral services and wider age ranges. Similarly, the possibility of new sources of vaccine that are more affordable can also change the landscape and enable vaccination for boys and older girls. On the other hand, if the new vaccines have sufficiently different characteristics from the current vaccines, there may be a need for countries to consider any trade-offs (for example, price versus efficacy versus impact) when making their selection of vaccines.

## 5 Conclusion

Although they have started later than wealthier countries in introducing HPV vaccine into national programs for a variety of reasons (LaMontagne et al., 2017), LMICs are beginning to catch up and are demonstrating that the barriers initially identified in the early years of HPV vaccine availability (from 2006 to 2012) are not insurmountable. HPV vaccine can be effectively delivered to young adolescent girls using a variety of strategies. While Gavi support has been instrumental in covering both vaccine and initial delivery costs in low and lower-middle income countries, upper-middle income countries face significant challenges in financing vaccine purchase. The prospect of new, more affordable vaccines entering the market may help overcome this hurdle, although there may also be other barriers or considerations that are holding some countries back (such as the low rates of cervical cancer in the Near East or a preference for using domestically produced vaccine).

While modeling has shown that HPV vaccination alone—even at 90% coverage of girls—cannot achieve elimination of cervical cancer in high-incidence regions like Sub-Saharan Africa and Latin America, which will also have the most difficulty developing robust screening and treatment programs, it can reduce incidence rates by at least half in just four decades (Brisson et al., 2020). Successful introduction and implementation of HPV vaccine into national programs is therefore a critical part of any elimination strategy. Recent progress is encouraging although challenges remain, and learnings from countries illustrate some options for preventing or overcoming these obstacles. There are opportunities to use the learnings to date and advances on the horizon to increase effectiveness and sustainability and move towards the coverage levels that are needed to achieve eventual elimination.

## Funding sources

PATH provides technical assistance for HPV vaccination programs in LMICs with funding provided by 10.13039/100001125Gavi, the Vaccine Alliance and collaborates with global partners in HPV vaccine policy with partial funding by the 10.13039/100000865Bill & Melinda Gates Foundation. The authors from PATH are responsible for the views expressed in this article and they do not necessarily represent the decisions, policy, or views of PATH, Gavi, the Vaccine Alliance, or the Bill & Melinda Gates Foundation.

## Disclaimer

Where authors are identified as personnel of the World Health Organization, the authors alone are responsible for the views expressed in this article and they do not necessarily represent the decisions, policy or views of the World Health Organization.

## CRediT authorship contribution statement

**Vivien D. Tsu**: Conceptualization, Writing - original draft, Writing - review & editing; **D. Scott LaMontagne**: Conceptualization; Data curation; Visualization; Writing – Reviewing and editing; **Phionah Atuhebwe**: Writing - review & editing; **Paul N. Bloem**: Writing - review & editing; **Cathy Ndiaye**: Writing - review & editing.

## Declaration of Competing Interest

The authors declare they have no conflicts of interest to report.
